# Flexible Intelligence on a Green Skeleton: Progress and Challenges of CNF-Enabled Multimodal Sensing Platforms

**DOI:** 10.3390/polym17212941

**Published:** 2025-11-03

**Authors:** Hemiao Wang, Guanlin Huo, Guijuan Xu, Dehai Yu, Shanshan Liu, Qiang Wang

**Affiliations:** 1 State Key Laboratory of Green Papermaking and Resource Recycling, Qilu University of Technology, Shandong Academy of Sciences, Jinan 250353, China; 10431240310@stu.qlu.edu.cn (H.W.); 10431230356@stu.qlu.edu.cn (G.H.); liushanshan8303@163.com (S.L.); 2 Weifang Huisheng Insulation Technology Co., Ltd., Weifang 261041, China; xuguijuan045@163.com

**Keywords:** cellulose nanofibrils, green flexible sensors, multiscale conductive networks, sustainable manufacturing, multimodal transduction

## Abstract

Cellulose nanofibrils (CNFs) provide a green scaffold for next-generation flexible sensors. They unite abundance, mechanical robustness, biocompatibility, and an easily engineered surface. This review synthesizes advances from the past five years in low-carbon CNF manufacturing. We cover biomass pretreatment, high-solid mechanical fibrillation, and in situ functionalization. We then elucidate mechanisms that govern CNF films, aerogels, and double-network hydrogels used across humidity, temperature, strain/pressure, optical, electrochemical, and biosensing platforms. Particular attention is given to multiscale conductive networks, surface-charge regulation, and reversible dynamic crosslinking. Together, these motifs raise sensitivity, widen the linear response windows, and strengthen environmental tolerance. We interrogate bottlenecks that impede scale-up, including energy demand, batch-to-batch variability, and device-level integration. We also assess prospects for deep-eutectic-solvent recycling, roll-to-roll digital printing, and algorithm-guided structural design. Finally, we outline directions for self-healing and self-powered biomimetic architectures, fully degradable life-cycle design, and integrated “sense–store–compute” nodes. These analyses chart a credible path from laboratory discovery to industrial deployment of CNF-based sensing technologies.

## 1. Introduction

Cellulose nanofibrils (CNFs) are fibrillar assemblies (typically ~5–60 nm in width and micrometer-scale in length) with surfaces rich in hydroxyl groups, whereas cellulose nanocrystals (CNCs) are highly crystalline, rod-like nanoparticles [1,2,3]. Alternating crystalline and amorphous segments confers outstanding mechanical robustness and architectural compliance, in marked contrast to the highly crystalline, rigid CNCs. The appeal of CNFs arises from their abundance (wood, bamboo, and agricultural residues), renewability, biocompatibility, and biodegradability [4]. In practice, virtually any cellulose-rich feedstock can serve as a precursor: beyond wood and common plant fibers, agricultural by-products, herbaceous fibers, certain algae, and bacterial cellulose have all been harnessed to produce CNFs [5]. With appropriate pretreatment and mechanical disintegration, diverse sources yield fibrils of comparable nanoscale dimensions and properties, ensuring a broad and resilient raw-material base for scale-up. Coupled with a high specific surface area, optical transparency, and a hydroxyl-rich surface that readily undergoes chemical modification, CNFs offer a versatile platform for advanced functional materials [6]. Entanglement and percolation of CNFs generate hierarchical networks that underpin these behaviors [7].

Guided by the tenets of sustainable development and catalyzed by rapid advances in nanomaterials, CNFs have become a focal point of contemporary research. In sensing science, its hierarchical nanoscale architecture, mechanical pliancy, optical transparency, and dense surface hydroxylation are decisive attributes [8]. Chemical routes—exemplified by TEMPO (2,2,6,6-tetramethylpiperidine-1-oxyl)-mediated oxidation—install carboxylates and other moieties on the fibril surface, generating oxidized CNFs (TOCNFs) with superior aqueous dispersibility and appreciable ionic conduction [9,10]. These features enable transduction schemes that rely on ion migration, interfacial proton transport, and dielectric modulation. The native –OH groups serve not only as handles for post-functionalization but also as adsorption and hydrogen-bonding sites for analytes. In humidity sensors, they accelerate water uptake and facilitate proton hopping within thin hydration layers, thereby enhancing signal strength and stability [11].

Beyond TEMPO chemistry, greener modification platforms continue to expand the toolbox. Deep eutectic solvents (DESs) can concurrently swell and esterify cellulose, yielding CNFs with high carboxylation, increased production efficiency, and improved thermal stability [12]. Such preparative and post-synthetic strategies broaden the physicochemical design space. They allow deliberate tuning of surface charge density, wettability, and network connectivity, which in turn supports the diverse requirements of humidity, temperature, strain/pressure, optical, electrochemical, and biosensing modalities. Several recent high-level reviews converge on this view. Cellulose-based materials are increasingly recognized as credible building blocks for green electronics and sensing, and their integration into device-level architectures is drawing sustained attention [5]. The use of cellulose to construct sensors across multiple signal domains has thus emerged as a prominent research thrust, with notable gains documented over recent decades [13].

The “green” attributes of CNFs—renewability and biodegradability—position them as a sustainable alternative to conventional synthetic materials in sensor development, addressing the escalating environmental burden of electronic waste [6]. Whereas traditional sensor platforms often rely on petroleum-derived polymers or energy-intensive inorganic components, CNFs originate from abundant biomass, and their biodegradability offers a route to mitigate end-of-life accumulation, aligning with global sustainability goals and motivating research into CNF-based sensors [14]. Notably, electronic-waste generation continues to climb, reaching approximately 62 million tons worldwide in 2022, yet less than one-quarter is formally recycled [15]. This disparity underscores the urgency of degradable and recyclable electronics. Relative to conventional materials, CNF-based sensors can be designed to degrade naturally or to be recycled at the end of their service life, thereby reducing electronic waste at source and improving environmental compatibility [15]. For example, recent work has demonstrated the feasibility of “green” electronics by using wood-derived nanocellulose to fabricate biodegradable printed-circuit-board substrates [15]. Such prototypes suggest that CNF-enabled devices can satisfy performance requirements while avoiding non-degradable residues at the end of life, informing the design of future environmentally responsible electronics.

Beyond end-of-life advantages, the intrinsic material performance of CNFs has elevated its status in sensing technologies [1]. CNF-based films combine high mechanical strength with dimensional stability, enabling their use as flexible substrates or active elements that integrate and support additional functional components, thereby enhancing device reliability and durability. In parallel, the high specific surface area and porous network of CNFs promote sensitivity to target analytes. For instance, the nanochannel architecture of CNF films can adsorb large quantities of gaseous or liquid molecules, allowing CNF-based humidity and gas sensors to deliver rapid and pronounced responses [12]. The abundance of surface hydroxyl groups facilitates straightforward functionalization, permitting the incorporation of conductive nanoparticles, chromophores, or biological receptors to engineer composite sensing media for temperature, strain, chemical, and biomolecular detection [6,16]. Collectively, these features endow CNFs with flexibility and multifunctionality for constructing next-generation sensors, exemplifying the integration of “functional materials” with “sustainable development” in nanomaterials science [1]. With ongoing advances in fabrication and post-synthetic modification, the prospects of CNFs in sensing and electronics continue to expand. To meet practical deployment needs, efforts now focus on improving large-scale production efficiency and batch-to-batch consistency. For example, one study reported a green, recyclable solvent system that achieves efficient separation and carboxylation of cellulose, markedly improving the production efficiency and quality of CNFs [12]. Such process innovations are expected to lower costs and the environmental footprint, accelerating the translation of CNF-based sensors.

**Figure 1:**
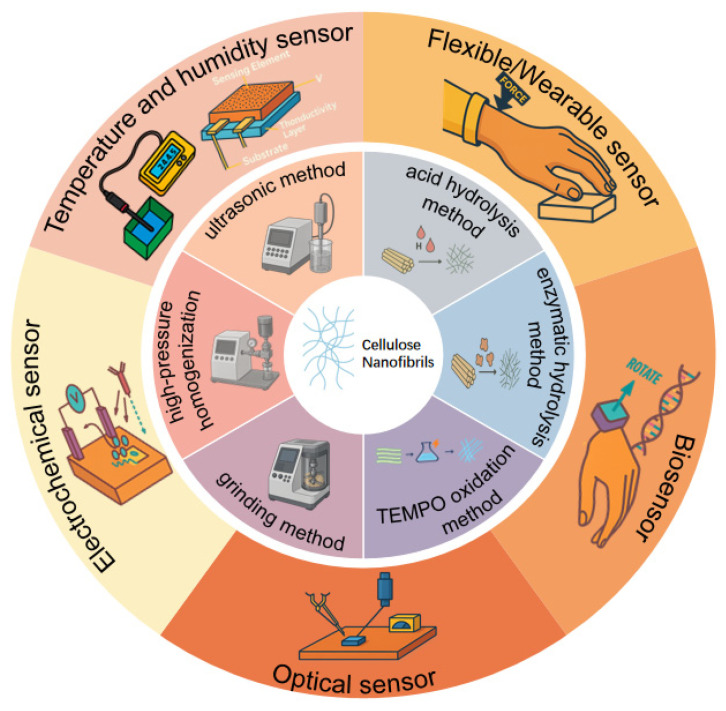
Graphical abstract.

Advances in CNF preparation and performance have, in turn, catalyzed broader adoption of bio-based materials in sensing. This review systematically summarizes the main preparation routes for CNFs and their diverse applications in sensors, as illustrated in Figure 1. Section 2 details mechanical, chemical, and other promising strategies for producing CNFs. Section 3 discusses the applications and roles of CNFs across sensing modalities. Together, these sections provide a comprehensive and coherent overview of CNF preparation and their utilization in sensing technologies. Specifically, our work distinguishes itself by (i) emphasizing sustainable and low-carbon CNF manufacturing routes (e.g., DES-, plasma-, and enzyme-assisted processes), (ii) systematically linking multiscale conductive network design with multimodal sensing performance, and (iii) outlining industrial translation strategies including roll-to-roll printing, self-healing, and fully degradable CNF-based devices—topics seldom discussed in earlier reviews. Table 1 contrasts bio-based platforms (CNFs and related biopolymers) with conventional synthetics. Bio-based routes offer renewable feedstocks, biocompatibility, aqueous/printing processes, and tunable surface charge and network topology for multimodal sensing; key trade-offs are humidity-driven drift and batch variability, mitigable via encapsulation, hydrophobization, and covalent filler interfaces. Synthetics provide out-of-the-box thermal/moisture robustness and mature QC but carry higher environmental burdens and e-waste. This comparison frames material selection and foreshadows the modality-specific CNF data summarized later.

## 2. Synthesis and Preparation of CNFs

### 2.1. Mechanical Disintegration Methods

#### 2.1.1. High-Pressure Homogenization (HPH)

**Figure 2:**
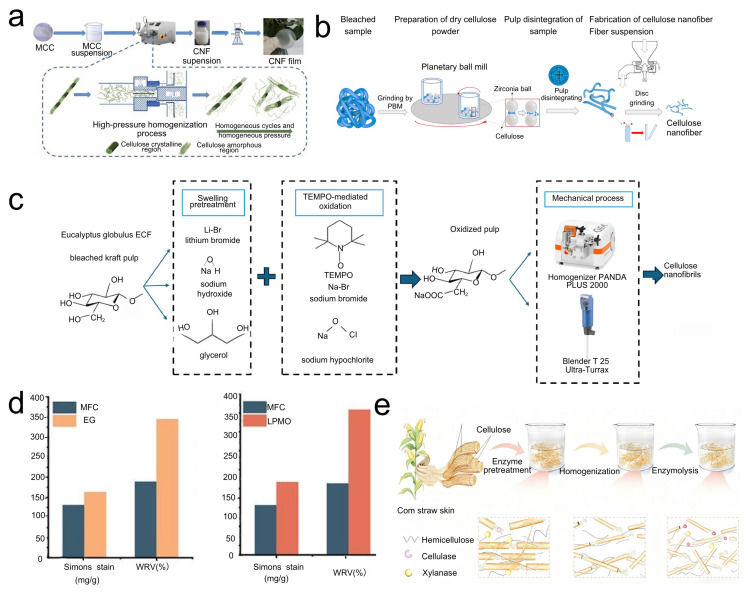
(**a**) Flowchart of CNF film preparation via vacuum filtration after CNFs are produced from microcrystalline cellulose through HPH. (**b**) Flowchart of CNF preparation using planetary ball milling. (**c**) Production scheme of CNFs, including chemical structures of the process and equipment information. (**d**) Effects of endoglucanase (EG) and lytic polysaccharide monooxygenase (LPMO) on the water retention value and pigment adsorption of microcrystalline cellulose. (**e**) Enzyme pretreatment/mild homogenization/enzymolysis strategy for the preparation of CNCs from corn straw pulp.

The principal routes to obtaining CNFs include mechanical, chemical, and composite methods, and the chosen approach strongly influences fibril morphology, properties, and production efficiency. Purely mechanical treatment of cellulose is time-consuming and energy-intensive; consequently, pretreatment is typically employed to improve process efficiency. HPH applies very high mechanical pressure to cellulose feedstocks via a high-pressure homogenizer, inducing fibrillation through shear, cavitation, and impact, thereby generating CNFs. Yao prepared CNFs with diameters of 10–20 nm from microcrystalline cellulose using HPH (Figure 2a shows the preparation flowchart) [10]. Li obtained CNFs with an average diameter of 27 nm by combining DES pretreatment with HPH, a strategy that can replace conventional pretreatments, shorten the process, improve pretreatment efficiency, and reduce environmental pollution [17]. Notably, recent research has focused on lowering the energy demand of HPH and enhancing its suitability for continuous production. For example, increasing the initial solid content of the cellulose slurry can substantially reduce the energy required for fiber dissociation per unit mass. Enzyme-assisted HPH operated using high solids (generally above 10%) has been shown to minimize excess water and improve energy efficiency [18]. In addition, introducing mild chemical media prior to homogenization markedly decreases energy consumption; for instance, a team at Oak Ridge National Laboratory (USA) reduced energy use by about 21% in a pilot-scale disk-mill–HPH process by adding trace NaOH/urea to the fiber slurry, and the resulting CNFs were comparable in crystallinity, morphology, and strength to those produced in water alone [19]. Such media weaken inter-fiber hydrogen bonding and interfacial cementation, thereby facilitating more efficient mechanical dissociation during HPH. Overall, by optimizing homogenization parameters and integrating suitable pretreatment media, HPH is trending toward lower energy consumption and larger-scale, continuous production [20].

#### 2.1.2. Grinding and Slurry-Making Technologies

Through mechanical impact and friction between grinding media (e.g., millstones or balls) and cellulose pulp, cellulose fibers are refined into nanofibers. Uranchimeg [21] reported that, relative to traditional grinding (Method A), planetary ball milling (Method B) yields more uniform CNFs with smaller fiber diameters (Figure 2b shows the process flow of CNF preparation by planetary ball milling). The energy consumption of commercial stone mills (SuperMassCollider™) is reported to be 5–30 kWh/kg [22]. In recent years, new low-energy grinding equipment and process refinements have emerged. For example, planetary ball milling combined with sequential grinding strategies has been used to produce highly crystalline CNFs, with specific energy consumption as low as ~2.95 kWh/kg—among the lowest values reported to date [23]. This indicates that efficient cellulose dissociation can be achieved without additional chemical or enzymatic pretreatment by optimizing ball-milling parameters, such as rotation speed, grinding time, and grinding sequence. In addition, twin-screw extrusion grinding, a comparatively new mechanical dissociation technology, has been reported to continuously prepare CNFs at high-solid contents of 20–30%, with energy consumption reduced by ~60% compared with traditional processes [24]. These advances suggest that, by developing continuous, low-energy mechanical dissociation equipment (e.g., customized disc mills, twin-screw extruders) and integrating process optimization, large-scale and efficient CNF production is achievable [20,25]. It is also noteworthy that unconventional mechanical dissociation methods—such as high-speed jet, supercritical fluid explosion, and cold plasma-assisted grinding—are being explored to obtain high-performance CNFs while lowering energy demand. For example, cold plasma or ultrasound can be coupled with grinding to accelerate fiber dissociation; another emerging approach employs non-polar “pseudo-solvent” media to promote spontaneous peeling of cellulose microfibrils, enabling nanofiber formation without high-intensity mechanical input. These strategies open new avenues for the mechanical preparation of CNFs.

#### 2.1.3. Ultrasonic Treatment

High-frequency ultrasound generates cavitation bubbles within cellulose suspensions. The collapse of these bubbles produces localized, high-temperature and high-pressure microenvironments—together with shock waves—that disrupt the cellulose microstructure, lower the degree of polymerization, and disperse agglomerates. Abdullah K. Alanazi [9] effectively fibrillated cellulose into nanofibers approaching elementary-fibril thickness using ultrasonic treatment. Ultrasound is frequently deployed as an auxiliary step alongside mechanical dissociation to enhance efficiency and reduce fiber dimensions. Compared with other purely mechanical methods, ultrasonic treatment typically consumes less energy but offers limited throughput per run, and is therefore mainly used at laboratory-scale. The intensity and duration of sonication strongly influence CNF length and crystallinity: moderate ultrasound promotes further exfoliation without substantive damage to crystalline domains, whereas excessive exposure can yield overly short fibers and reduced crystallinity [26]. Accordingly, practical implementation requires a careful balancing of ultrasound intensity and duration to obtain CNFs with appropriate aspect ratios and well-balanced properties.

### 2.2. Chemical and Chemical–Mechanical Methods

#### 2.2.1. TEMPO-Mediated Oxidation

Chemical treatments aim to modify cellulose or remove bound components to facilitate fibrillation, often yielding CNFs with defined surface chemistries [9,10]. TEMPO radicals, used with NaBr and a primary oxidant (e.g., NaClO), selectively oxidize the C6 primary hydroxyls of cellulose to carboxyl groups under alkaline conditions (pH ~10) [27]. The resulting carboxylate functionality imparts negative charge and inter-fibril electrostatic repulsion, markedly promoting separation into individual nanofibers under mild mechanical treatment (e.g., stirring and light homogenization) [27,28]. Tang [29] reported that periodic NaClO dosing produced nanofibers with a degree of polymerization 2.3 times higher than that obtained with a single NaClO addition, providing a feasible route to nanofibers with high carboxyl content and a controlled, high degree of polymerization [28]. TEMPO oxidation is, thus, both an efficient route to CNFs and a means of installing carboxyl groups, as illustrated in Figure 2c [30]. These groups are pivotal for sensing, enabling, for example, covalent immobilization of biological receptors and the fabrication of pH-responsive materials [31]. The central challenge is to balance oxidation efficiency while minimizing cellulose degradation and costs [28]. Carboxyl groups furnish reaction sites and confer negative charge that can be leveraged for electrostatic interactions with analytes or other materials. Because oxidation predominantly targets accessible fibril surfaces, it promotes dissociation [32]; however, excessive oxidation can cleave glycosidic bonds and weaken CNFs [33].

#### 2.2.2. Acid Hydrolysis for CNF Production (Distinct from CNC Production)

Whereas strong acid hydrolysis under harsh conditions typically yields CNCs by removing amorphous regions completely, milder acid treatment coupled with mechanical action can produce CNFs [34]. The objective is controlled, partial hydrolysis and swelling to facilitate fibrillation [35]. Atiqah employed low-concentration oxalic acid for mild hydrolysis following supercritical CO_2_ pretreatment, obtaining CNFs with widths of 10–15 nm. Oxalic-acid treatment produces CNFs with strong gel-forming ability, amphiphilicity, and electrostatic repulsion, and is considered more environmentally friendly owing to straightforward recovery. Reported oxalic-acid CNF widths are 6–20 nm, with lengths of 300 nm to 1.2 µm [36]. Formic acid, in combination with an FeCl_3_ catalyst, has also been used to produce CNFs from sugarcane bagasse; the yield decreases with increasing hydrolysis time [37,38]. Phosphorylation of cellulose via a phosphoric acid/urea system introduces phosphate groups, increases surface charge, and assists fibrillation, serving as an effective pretreatment for CNF production. The choice of acid and reaction conditions is, therefore, pivotal in determining whether the product is CNFs or CNCs [34]. For CNFs, hydrolysis must be controlled to weaken inter-fibril bonding without fully degrading amorphous segments. By contrast, strong acids such as sulfuric or hydrochloric acid preferentially erode amorphous regions at high concentrations and temperatures, producing CNCs [36]. Weaker organic acids or milder conditions can induce surface modification or partial hydrolysis, thereby reducing the energy required for subsequent mechanical fibrillation to yield CNFs [39].

#### 2.2.3. Enzymatic Hydrolysis

Cellulase enzymes—typically endoglucanase, sometimes acting synergistically with exoglucanase and cellulose-binding enzymes—can selectively hydrolyze amorphous regions or weaken fiber structures, thereby facilitating mechanical dissociation [40]. Endoglucanase randomly cleaves internal bonds within amorphous domains [41]. Ribeiro [42] combined enzymatic hydrolysis with ultrasonic treatment to obtain CNFs with a diameter of 180 nm and a crystallinity of 78%. Ramírez Brenes [43] produced CNFs of 250–300 nm with a crystallinity of 80.9% using a similar enzyme–ultrasound combination. Enzymatic hydrolysis is a “green” process, characterized by mild conditions and high specificity, and it can reduce energy consumption in subsequent mechanical steps [44]. However, enzyme cost and stability, extended reaction times, incomplete hydrolysis, the need for pretreatment to remove lignin/hemicellulose, and the subsequent mechanical processing [45] often result in low yields—frequently <20% in many studies—although pretreatment can improve these outcomes [46] (see Figure 2e for the enzyme pretreatment/gentle homogenization/enzymatic hydrolysis preparation method). Enzymatic hydrolysis provides a gentler pathway to CNFs, potentially retaining a higher degree of polymerization than some chemical routes [40]. The synergy among cellulase types and their coupling with mild mechanical action is critical for optimizing CNF yield and the properties [47].

Enzymes are highly specific catalysts that target defined bonds in cellulose; EG opens the fiber structure, making it more susceptible to further enzymatic attack or mechanical refining. Single-component enzyme treatment with LPMO improves the accessibility of microfibrillated cellulose produced from mechanically refined kraft pulp, as shown in Figure 2d, where enzyme-treated cellulose exhibits increased accessibility [40]. Cellulases are highly specific, targeting particular sites along cellulose chains: endo-enzymes loosen the internal fiber architecture, rendering it more amenable to subsequent enzymatic or mechanical separation [43]. Recent studies show that optimizing enzyme dosage and reaction time can effectively fine-tune the structural properties of the resulting CNFs. For example, Las-Casas and Arantes applied a single-step, low-dose endoglucanase pretreatment to wood pulp followed by mechanical ultrafine grinding, producing high-performance CNF films with ~50% lower mechanical energy consumption [20]. CNF films derived from enzyme-pretreated samples exhibited higher visible-light transmittance and markedly enhanced barrier properties relative to untreated controls, with reduced surface wettability and lower moisture uptake, while maintaining comparable mechanical strength and thermal stability. These results indicate that enzymatic regulation can save energy and cost while preserving CNF performance, offering an attractive route for industrial-scale production of high-quality CNFs. Notably, enzymatic hydrolysis can function not only as a pretreatment for fiber dissociation but also as a post-treatment after mechanical processing to further shear long fibers, reduce viscosity, and narrow particle-size distribution [48]. For example, adding a small amount of cellulase to coarse CNFs obtained by mechanical pulping reduced viscosity and narrowed the size distribution of the CNF suspension, which benefits subsequent film processing [3]. With advances in enzyme engineering and biocatalysis, the efficiency of enzymatic routes to CNFs is expected to improve further, underscoring their advantages for green, low-carbon production.

### 2.3. Other Promising Preparation Strategies

Composite strategies—such as in situ oxidation–mechanical routes—have been shown to markedly reduce the energy demand of CNF preparation. For example, Oxone pretreatment enhances the efficiency of HPH, lowers energy consumption, and serves as a strong complement to TEMPO oxidation [49]. In another case, sodium persulfate combined with UV (Ultraviolet) irradiation followed by HPH promotes cellulose dissociation, yielding smaller, more uniform CNFs and improving the transparency and mechanical strength of the resulting films [50]. Collectively, these composite methods exemplify a trend towards coupling chemical pre-oxidation with mechanical fibrillation to synergistically increase production efficiency and final performance. Oxidative pretreatment lowers the energy required for subsequent mechanical dissociation by partially disrupting fiber structure or introducing surface charges; for instance, UV light can accelerate specific oxidation reactions, introducing radical defects that weaken fibers [31,50]. Reducing mechanical energy input not only decreases consumption and costs but may also minimize over-grinding damage to the fibril structure, thereby producing CNFs with superior properties [30,51]. Beyond these approaches, a number of new green pretreatment and dissociation strategies have recently emerged and attracted broad interest. The following sections introduce several promising methods—including DESs, plasma, and microwave pretreatments—together with recent advances concerning the influence of raw-material sources on CNF performance, process scale-up, and structural regulation.

#### 2.3.1. DES Pretreatment

DESs are low-melting mixtures of hydrogen-bond donors and acceptors, characterized by low volatility, low toxicity, and tunable viscosity, and are regarded as green alternatives to conventional organic solvents and ionic liquids [25]. In CNF preparation, DESs can serve as a pretreatment medium to swell fibers and partially remove non-cellulosic components from lignocellulosic feedstocks, thereby lowering the difficulty of subsequent mechanical dissociation. In recent years, diverse DES systems (e.g., choline hydroxide/amino-acid salts and quaternary ammonium salts/amines) have been applied to pretreat lignocellulosic fibers and agricultural residues with promising outcomes. For example, Baraka et al. employed a trimethylammonium chloride (TEMA)–imidazole DES combined with microwave heating to pretreat flax fibers. Under optimized conditions, the hemicellulose and lignin contents decreased markedly, the crystallinity index increased, and the diameter distribution of the CNFs obtained after mechanical dissociation became more concentrated [48]. This result indicates that DES pretreatment can selectively dissolve amorphous components and reinforce the crystalline framework of cellulose, facilitating the production of uniformly sized nanofibers [48]. As another example, Ma et al. used a DES comprising choline hydroxide as the hydrogen-bond donor and L-proline hydrochloride as the acceptor to directly treat rice-husk fibers, producing cellulose nanofibers in a single step and combining them with graphene oxide to fabricate flexible supercapacitor electrodes [52]. This approach avoids traditional multistep workflows such as alkali treatment and bleaching, achieving green and efficient biomass utilization.

Notably, DESs not only swell and partially dissociate fibers during pretreatment but can also introduce specific functional groups. For instance, treating bamboo pulp with an oxalic-acid–choline-chloride DES can simultaneously drive esterification and introduce a small number of carboxyl groups, thereby enhancing the dispersibility and stability of the resulting CNFs [53]. In addition, many DES systems can be recovered and reused, further improving process sustainability [53]. In summary, DES pretreatment offers a versatile platform for the green production of cellulose nanofibers. By rationally designing DES composition and ratios for different feedstocks, targeted pretreatment can be achieved while reducing energy consumption and pollution and simultaneously regulating CNF surface chemistry and the structural attributes. The development potential of this field has been recognized by numerous recent reviews [54].

#### 2.3.2. Plasma Pretreatment

Cold plasma—particularly dielectric barrier discharge (DBD) plasma—is increasingly used as a surface-modification technique for biomass-fiber pretreatment, distinguished by the absence of a liquid phase or added chemical reagents. High-energy electrons, ions, and reactive species generated in the plasma bombard cellulose-fiber surfaces, breaking bonds and introducing functional groups; this etches the surface, reduces the degree of polymerization, and partially oxidizes the fibers [55]. Anari et al. extracted micro/nanocellulose by treating dried walnut-shell powder with atmospheric-pressure DBD plasma, followed by mechanical grinding [55]. Although plasma treatment slightly reduced cellulose extraction yield (from ~26% to ~22%), the treated fibers dissociated more readily, and the resulting nanofibers exhibited ~15% smaller diameters and reduced lengths relative to the untreated group [56]. X-ray photoelectron spectroscopy (XPS) showed increased oxygen-containing functionalities (e.g., C=O, O-C-O) on the fiber surface after plasma exposure, indicating enhanced hydrophilicity and reactivity that benefit subsequent dissociation and dispersion.

In a separate study, Zhu et al. employed Fe^2+^-assisted cold plasma to pretreat pineapple-peel fibers, followed by high-speed grinding to produce CNFs [16]. The combined Fe^2+^/CP plasma treatment markedly lowered the cellulose degree of polymerization and increased surface carbonyl content [16]; compared with untreated pineapple peel, the pretreated fibers yielded smaller-diameter nanofibers and higher production under identical mechanical conditions [16]. The advantages of plasma pretreatment include its dry, rapid, and waste-free nature, making it suitable for integration into industrial workflows. Current limitations include treatment non-uniformity for thicker fiber bundles and scale constraints imposed by discharge-device dimensions. Future work is likely to focus on roll-to-roll plasma systems capable of continuously processing fiber sheets or mats and coupling seamlessly with mechanical dissociation. Overall, plasma pretreatment provides a promising route to improve CNF production efficiency from non-wood and waste biomass, with minimal chemical consumption and environmentally friendly characteristics, aligning with the goals of sustainable development [55].

#### 2.3.3. Microwave-Assisted Technology

Microwave heating, owing to its rapid and volumetric energy delivery, has been used to enhance cellulose pretreatment and dissociation. On the one hand, microwaves rapidly heat water and reagents within fibers to elevated temperatures and pressures, thereby promoting cellulose swelling and hydrolysis. On the other hand, microwave irradiation can partially degrade components such as lignin, reducing fiber rigidity. Many green pretreatments exhibit markedly improved efficacy when coupled with microwaves. For example, integrating microwaves into the aforementioned DES pretreatment substantially shortens processing time and increases the removal efficiency of hemicellulose/lignin [48]. Baraka et al. showed that DES–microwave pretreatment increased the thermal stability and crystallinity of cellulose fibers while lowering the energy demand of subsequent mechanical dissociation [48]. As another example, Sadeghi-Shapourabadi et al. employed microwave-assisted alkali/hydrogen peroxide for chemical purification of potato-peel waste, followed by TEMPO oxidation–ultrasonication, to produce nanocellulose with diameters of only 4–22 nm and crystallinity as high as 70% [57]. Compared with conventional heating, microwaves significantly shorten the time required for alkali treatment and bleaching, making cellulose purification more efficient.

Microwaves can also enhance enzymatic pretreatment. Intermittent microwave input during enzymatic hydrolysis has been used to accelerate enzyme adsorption and action on cellulose, thereby improving saccharification and fiber-dissociation efficiency [57]. It is important to note that excessive microwave exposure may degrade cellulose chains, causing a substantial decrease in degree of polymerization (DP); thus, microwave power and duration should be optimized. In parallel, studies show that increasing the number of mechanical microfluidization cycles markedly reduces CNF diameter and narrows size distribution, thereby improving the mechanical strength and barrier properties of the resulting films. However, introducing a phosphoric-acid esterification pretreatment prior to microfluidization—while imparting flame retardancy and other functionalities—slightly reduces fiber crystallinity [18]. Overall, microwave assistance provides an energy-saving, time-efficient means for cellulose pretreatment, particularly when combined with DESs, dilute acid, or alkali routes.

Purely mechanical routes remain the most energy-intensive: commercial stone/grinding mills report specific energy in the range of ~5–30 kWh·kg^−1^ [22], whereas optimized planetary ball milling reports values as low as ~2.95 kWh·kg^−1^ [23], demonstrating that mechanical-parameter optimization alone can substantially lower the energy demand. Emerging continuous mechanical approaches (e.g., twin-screw extrusion) report an ≈60% reduction in energy consumption compared with traditional grinding [24], attributable to higher solid processing and continuous operation. Hybrid/pretreatment strategies provide notable energy savings by weakening inter-fiber bonds before mechanical fibrillation: enzyme pretreatment has been reported to reduce subsequent mechanical energy consumption by ~50% [20], and pilot-scale chemical media (trace NaOH/urea) combined with disk-mill–HPH showed an ~21% energy reduction versus water-only processing [19]. Ultrasonic treatment is energetically modest at laboratory-scale (lower per-run energy than high-intensity mechanical routes) but suffers from limited throughput. Chemical routes (e.g., TEMPO-mediated oxidation, DES-assisted pretreatment) typically trade reagent/operational cost for lower mechanical energy: TEMPO oxidation enables fibrillation under milder mechanical conditions (thus reducing mechanical input) [32], whereas the combined DES/microwave pretreatment enables the production of CNFs with a more uniform fiber diameter distribution [48]. Plasma pretreatment and microwave-assisted methods likewise increase the ease of fibrillation (improved yield or smaller diameters under identical mechanical work) and, therefore, reduce effective energy per unit CNF, even if the exact kWh·kg^−1^ varies with setup [16]. Importantly, reported metrics vary widely with feedstock, solid content, equipment scale, and target CNF properties.

## 3. Applications of CNFs in Advanced Sensing Platforms

### 3.1. CNF-Based Temperature and Humidity Sensors

**Figure 3:**
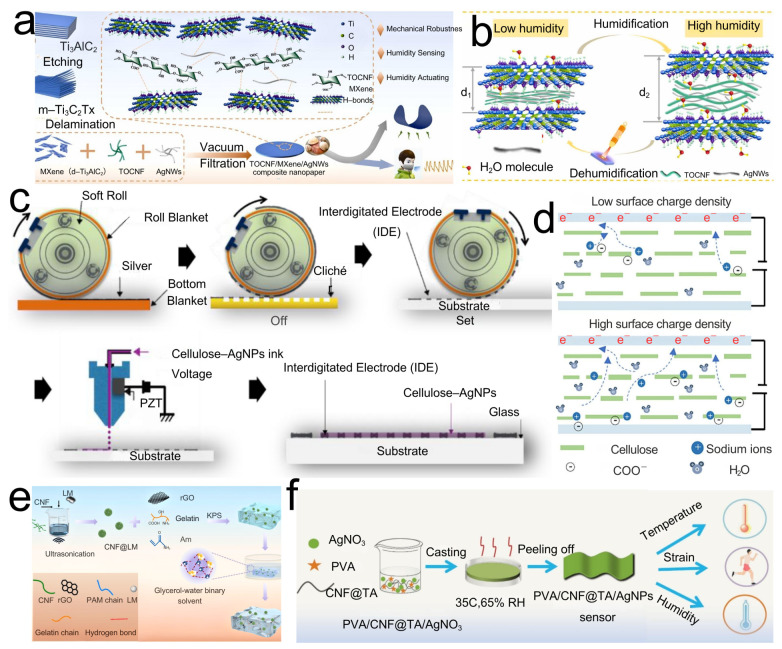
(**a**) Schematic diagram of the preparation method of TOCNFs/MXene/AgNWs and the sensing process. (**b**) Schematic diagram of the sensing mechanism of the temperature sensor. (**c**) Synthesis route of CNF-AgNPs and preparation flowchart of the humidity sensor. (**d**) Schematic diagram of the sensing mechanism of the surface-charge-regulated CNF film sensor. (**e**) Schematic representation of the synthesis of the CNF@LM/PAM/rGO/gelatin/glycerol (CLPRGG) hydrogel. (**f**) Preparation process and sensing application schematic of PVA/CNF@TA/AgNPs.

The distinctive attributes of CNFs—high specific surface area, mechanical flexibility, tunable surface chemistry, and intrinsic hydrophilicity—make them an ideal material for diverse sensing applications. Humidity sensing is readily realized with CNFs, particularly TOCNFs bearing carboxyl groups that confer ionic conductivity. Adsorbed water promotes ion migration, producing measurable changes in resistance or impedance. The Grotthuss mechanism (proton hopping) is pertinent: water’s high dielectric constant and its uptake into the CNF matrix alter the composite dielectric constant, thereby modulating capacitance [58]. In CNF/TOCNF humidity sensors, adsorbed water first forms hydrogen-bonded layers on hydroxyl/carboxylated surfaces; protons then migrate by the Grotthuss mechanism along these H-bond networks. As the RH increases, multilayer water and capillary condensation generate percolating aqueous pathways. Because the dielectric constant of water greatly exceeds that of dry CNFs, the effective permittivity (ε_eff_) of the CNF/water composite rises with water content and interfacial polarization so the device capacitance $C=\varepsilon_{0}\varepsilon_{\mathrm{eff}}A/d$ increases with RH. Upon desorption, disruption of H-bond networks and shrinkage of water pathways lower ε_eff_ and the capacitance, explaining the reversible capacitance–humidity response observed in CNF-based sensors. The hydrophilicity of CNFs (abundant –OH groups) facilitates water uptake, while the porous three-dimensional network of CNF films or hydrogels provides an extensive interfacial area for water–matrix interactions, enhancing sensitivity [59]. Han [60] reported a composite nanopaper comprising CNFs, MXene, and AgNWs, fabricated by vacuum filtration (see the preparation schematic in Figure 3a). This material integrates flexibility, conductivity, and mechanical robustness, delivering excellent humidity-sensing performance and intelligent actuation (see the sensing schematic in Figure 3b). The mechanism relies on adsorption/desorption of water within the high-surface-area conductive network, which perturbs percolation pathways and yields impedance responses. Across 11–97% RH, the sensor exhibits rapid response (<1 s) and recovery (<4 s), strong linearity (R^2^ > 0.99), and cyclic stability (no performance degradation after 1000 bends). The same construct functions as a humidity-driven actuator with pronounced, reversible deformation, indicating promise for flexible electronics, wearables, and paper-based smart devices.

Mijin Won [61] developed a fully printed cellulose nanofiber–silver nanoparticle (CNF-AgNP) composite for high-performance humidity sensors, highlighting the potential for sustainable flexible electronics. Using reverse-offset printing, renewable CNFs serve as the matrix and AgNPs as conductive fillers. Variations in humidity modulate internal ionic conductivity, producing a resistive response (the CNF-AgNP synthesis route is shown in Figure 3c). Within 10–90% RH, the device exhibits excellent sensitivity, rapid response (≈4 s) and recovery (≈34 s), good repeatability, and long-term stability, suitable for environmental humidity monitoring. Owing to their flexibility and printability, such sensors can be integrated directly onto paper or plastic substrates, enabling applications in smart packaging, wearable devices, and environmental monitoring—demonstrating green, low-cost, high-performance pathways for CNF-based humidity sensing. Zhu [62] designed a standalone, high-strength, highly sensitive CNF-film humidity sensor by modulating surface charge density (surface-charge-regulation CNF-film sensor performance schematic shown in Figure 3d). At a surface charge density of 1.45 mmol g^−1^, the sensor shows strong linearity over 15–75% RH and a high humidity sensitivity of 44.5% (%RH). The device can detect human humidity and locate water-leakage points, underscoring the broad potential of nanocellulose films in humidity sensing.

Temperature sensing typically exploits changes in conductivity/resistance of CNF composites containing thermosensitive components or dimensional variations with temperature. Zou [63] used CNFs and liquid metal to initiate acrylamide polymerization as the conductive phase, with gelatin and graphene oxide added to enhance mechanical properties, electromagnetic shielding, and conductivity. Crosslinking in glycerol yielded a hydrogel with antifreeze and water-retention properties (schematic diagram of CLPRGG hydrogel synthesis shown in Figure 3e). The resulting hydrogel exhibits high tensile strength, self-adhesion, wide environmental tolerance, electromagnetic shielding, and antibacterial activity. It is temperature-sensitive with a response time of 210 ms and recovery of 150 ms, and also strain-sensitive, capable of monitoring human motion and body-temperature changes at room temperature and under extreme conditions. Gong [64] employed poly(vinyl alcohol) (PVA) as the substrate, with tannic-acid-modified CNFs improving compatibility with PVA and enhancing mechanical properties. In situ reduction generated silver nanoparticles to form conductive channels. The construct demonstrates high sensitivity to body-temperature variations and a rapid thermal response, enabling real-time temperature monitoring (PVA/CNF@TA/AgNPs preparation flowchart shown in Figure 3f). In extended applications, CNFs underpin conductive flexible sensors with excellent electromechanical responsiveness, fast response, and good reversibility, allowing the detection of multiple stimuli such as pressure, and suiting wearable devices, health monitoring, and smart interfaces. This strategy is green, energy-efficient, and highly tunable, offering practical routes to functional integration and sensor design, and illustrating the wide prospects of sustainably produced nanocellulose in flexible electronics.

### 3.2. Flexible and Wearable Sensors Based on CNFs

**Figure 4:**
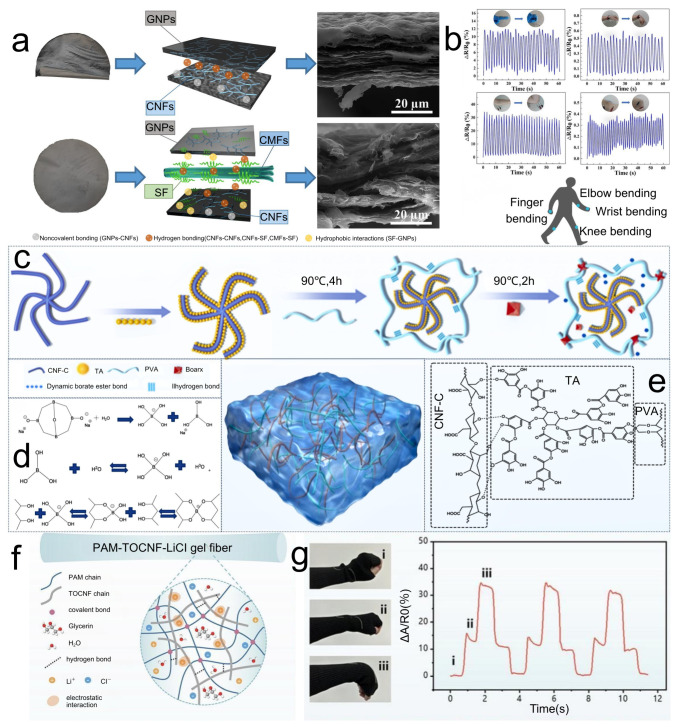
(**a**) Flowchart of the preparation process for flexible sensors. (**b**) Schematic diagram of the application of flexible sensors in human body sensing. (**c**) Synthesis route of PVA-CNF-C composite hydrogel. (**d**) Process of dynamic borate bond formation. (**e**) Structural formula of the hydrogel. (**f**) Internal structure and chemical composition of PAM-TOCNF-LiCl conductive gel. (**g**) Real-time resistance change in the ultra-stretchable properties of PAM-TOCNF-LiCl.

Deformation of a CNF-based conductive network alters the percolation pathways and, consequently, resistance [65]. In capacitive modes, deformation changes the inter-electrode spacing or the effective dielectric constant of CNF-based materials, thereby modulating capacitance. CNFs provide flexibility, mechanical reinforcement, and an effective dispersing matrix for conductive fillers—including graphene, carbon nanotubes, MXene, AgNWs, and liquid metals—while also supporting ionic conductivity in hydrogel-based sensors. Li [66] prepared a composite paper by casting micro/nanocellulose, silk fibroin, and high-load graphene. Synergy between micro/nanocellulose and silk fibroin afforded flexibility, electrical conductivity, and high mechanical strength. Silk fibroin promoted the tight coating of graphene nanosheets on the microcellulose surface, enabling integration into smart wearable biosensors embedded in garments (Figure 4a,b). Liu and colleagues [67] uniformly blended carboxylated CNF and tannic acid in PVA, then introduced aluminum ions to form a composite hydrogel. Aluminum ions strengthened tension and elasticity within the crosslinked network, enhancing mechanical adaptability and sensitivity. The hydrogel exhibited high mechanical performance, good conductivity, and strong responsiveness, though elevated aluminum-ion concentrations may present biological toxicity concerns (Figure 4c–e). Li [68] fabricated a double-network hydrogel using TOCNFs and polyacrylamide via a solvent-free, template-assisted strategy. Incorporation of TOCNFs markedly improved fiber fracture strength, strain, and toughness. Addition of lithium chloride and glycerol imparted dehydration resistance, frost resistance, and robust ionic conductivity. The gel fibers showed excellent transparency and elasticity with mechanical metrics of fracture strength, strain, and toughness reaching 3.55 MPa, 1715.66%, and 4.75 MJ m^−3^, respectively; an ionic conductivity of 0.128 S m^−1^; a linear strain-sensing response (gauge factor, GF, of 0.8128); and strong resistance to dehydration and freezing, ensuring stable performance across environments for the real-time monitoring of human motion (Figure 4f,g).

### 3.3. CNF-Based Biosensors

**Figure 5:**
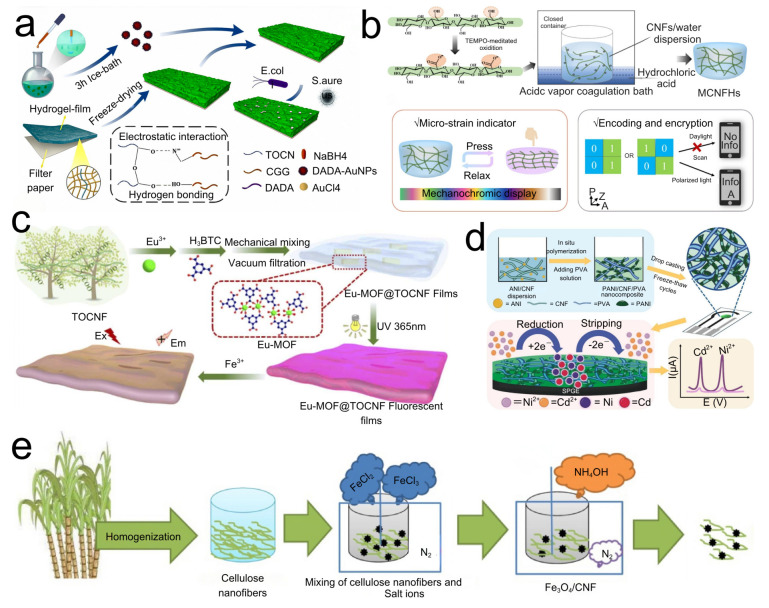
(**a**) Flowchart of the biosensor fabrication process where DADA-AuNPs are encapsulated on filter paper modified with CGG composite hydrogel. (**b**) Preparation and application of mechanochromic MCNFHs. (**c**) Schematic diagram of the preparation process for Eu-MOF@TOCNF fluorescent film. (**d**) Schematic diagram of the electrochemical reaction process. (**e**) Diagram relative to synthesis procedure of Fe_3_O_4_/CNF.

Biosensors integrate a biological recognition layer with a physicochemical transduction layer and electronic-signal-processing module, providing analytical tools for detecting analytes across diverse environments [69]. Dai [70] developed a paper-based colorimetric biosensor by encapsulating D-alanyl-D-alanine-capped gold nanoparticles (DADA-AuNPs) on filter paper modified with a TOCNF/cationic guar gum (CGG) composite hydrogel (preparation flowchart Figure 5a). Smaller DADA-AuNPs underwent faster color transitions upon bacterial contact, and higher concentrations of Staphylococcus aureus produced correspondingly quicker color changes. The device detected bacterial loads as low as 10 CFU mL^−1^ within 15 min, underscoring its promise for simple, rapid bacterial screening. Laila Hossain [71] and colleagues prepared a TOCNF hydrogel by coating and drying a cellulose gel containing glucose oxidase and phenol red onto a paper substrate. The nanocellulose gel extended the functional lifetime and enhanced the usability of glucose oxidase. Embedding a pH indicator within the nanocellulose hydrogel enabled a red-to-orange shift driven by the pH decrease arising from enzymatic production of gluconic acid and hydrogen peroxide. This platform detected glucose concentrations of 7–13 mM over 4–40 °C, demonstrating that nanocellulose-based sensors can support biomedical diagnostics by tailoring enzyme systems to specific diseases.

### 3.4. Optical Sensors Based on CNFs

Owing to its high transparency and flexibility, nanocellulose film is well-suited as a substrate for flexible optical sensing. Nonetheless, intrinsic size variability and the challenge of achieving controlled CNF orientation present obstacles for intelligent optical systems. Addressing straw burning and heavy-metal pollution, Vijay Kumar [72] isolated CNFs from rice straw and grafted L-histidine to form a Schiff base, endowing the CNFs with fluorescence for detecting Cr and Hg in water. The sensor exhibited high selectivity and a lower detection limit than many nanomaterial analogs. It also formed complexes with transition metals and retained reusability, with fluorescence intensity decreasing by only 33% after four cycles, thereby enabling high-value utilization of straw and laying the groundwork for cellulose-based wastewater treatment. Yu [73] prepared mechanochromic aerogels (MCNFHs) via TEMPO-mediated oxidation of CNFs followed by hydrochloric-acid vapor diffusion, which promoted physical crosslinking between CNFs by neutralizing surface carboxyls. The resulting MCNFHs displayed solvent-dependent mechanochromism, with pronounced color changes from yellow to red, blue, and green (Figure 5b shows the preparation and application of mechanochromic MCNFHs). Wang [74] synthesized a uniform Eu(III)-based metal–organic framework (Eu-MOF) and grew it in situ on TOCNFs, yielding Eu-MOF@TOCNF fluorescent films by vacuum filtration (Figure 5c depicts the preparation process). These films responded rapidly to Fe^3+^ in water, with fluorescence intensity showing a significant linear dependence on Fe^3+^ concentration. Quan [75] developed a biomimetic cellulose nanofiber-based double information encryption sensor (CNF-DIES) based on acetylated cellulose nanocellulose (ACNF), employing fluorescein isothiocyanate and protoporphyrin IX-modified ACNF as a pH-responsive switch. Under UV light, the fluorescence color shifted from red to green as pH increased; between pH 3 and 11, the fluorescence-intensity ratio varied linearly with pH. CNF-DIES exhibited strong stability and concealability and could be used directly as an ink. CNF-DIES films enabled fingerprint imaging, indicating broad potential in secure information transmission and forensic analysis.

### 3.5. CNF-Based Electrochemical Sensors

Electrochemical sensors are recognized for their high sensitivity and practical advantages, including simple instrumentation, short analysis times, good reproducibility, and compatibility with online monitoring—features that point to strong growth potential [76,77,78]. 4-Chlorophenol (4-CP), widely used in dyes, preservatives, pesticides, and herbicides, is highly toxic; hence, the rapid quantification of 4-CP in complex samples is essential [79,80]. Deng [81] incorporated Fe_3_O_4_ into CNFs (Fe_3_O_4_/CNF) as an amplifier for carbon-paste electrode (CPE) modification (Figure 5e displays the synthesis procedure of Fe_3_O_4_/CNF). The Fe_3_O_4_/CNF/CPE sensor delivered a linear response to 4-CP over 1.0 nM–170 μM with a detection limit of 0.5 nM, indicating high sensitivity. The optimal catalyst loading was 8.0 mg Fe_3_O_4_/CNF and the optimal pH was 6.0. Under these conditions, the 4-CP oxidation current increased by >1.67-fold relative to a bare CPE and the oxidation potential shifted negatively by ~120 mV. The sensor showed high recoveries in real-sample analyses, supporting its utility for environmental monitoring and public-health protection.

Heavy-metal contamination in industrial wastewater poses significant risks to human health [82,83]. Cadmium (Cd^2+^) and nickel (Ni^2+^) can damage vital organs and the respiratory system, respectively; therefore, rapid, reliable determination of heavy-metal content is critical. Boonkrong [84] developed a polyaniline/cellulose nanofiber/PVA hydrogel-modified screen-printed graphene electrode (PANI/CNF/PVA/SPGE) that enabled simultaneous detection of Cd^2+^ and Ni^2+^ and was successfully applied to real industrial-wastewater samples (see the preparation flowchart in Figure 5d). Incorporation of CNFs introduced a porous scaffold and favorable dispersion of electrode materials, enhancing the hydrogel’s sensing performance and highlighting the promise of porous conductive hydrogels for improved electroanalytical environmental monitoring. Xanthine (XA) monitoring is essential for assessing fish freshness, wherein xanthine oxidase (XO) is used to quantify XA as an indicator of spoilage [48]. Joyati Das [85] prepared CNFs from raw cotton via sulfuric-acid hydrolysis, cast CNFs onto a glassy-carbon electrode [34], and covalently immobilized XO on glutaraldehyde-activated CNF-modified GCE. The sensing mechanism couples enzymatic production of H_2_O_2_ with its subsequent electrochemical oxidation, yielding a current proportional to XA. The resulting biosensor achieved a lower detection limit than previously reported devices, with CNFs providing a high specific surface area for XO immobilization and reducing overall sensor cost—offering a promising material platform for fish-freshness evaluation.

Table 2 details recent material structures, structural forms, and significant performance parameters of CNF-based sensors, underscoring the fact that employing CNFs as a structural “skeleton” to develop composites including different functional components has become an essential strategy of enhancing sensitivity and environmental versatility. In relative-humidity/temperature sensing, TOCNF-MXene-AgNW and CNF-AgNP inkjet films achieved rapid responses of 1 s through the establishment of continuous conductive networks utilizing two-dimensional conductive films and metal nanoparticles, respectively [60,61]. Zhu et al. [62] remarkably extended sensitivity to 44.5%/%RH by tailoring carboxyl-group densities on TOCNF surfaces, suggesting interfacial charge-engineering amplification of proton-migration processes. For strain/pressure sensing, liquid-metal- or AgNPs-PVA-filled CNFs form antifreeze hydrogels and multifunctional, flexible films showing constant conductance at −40 °C and transient responses of <100 ms [63,64]. Micro/nanocellulose–silk-fibroin–graphene composite papers and tannic-acid-Al^3+^ crosslinked hydrogels [66,67] demonstrate that the establishment of multiple energy-dissipation networks through hydrogen bonding and metal coordinate bonds can significantly improve the linear range of detection whilst retaining high GF values.

The bio/optical sensing area highlights the unique value of CNFs for biomolecule immobilization and visual readouts. The paper-based TOCNF-CGG-DADA-AuNPs sensor identifies 10 CFU mL^−1^ bacteria in 15 min [70], and the TOCNF–Eu-MOF fluorescent film displays quick Fe^3+^-induced quenching, disclosing the synergistic recognition–amplification effect of MOF and CNFs [74]. L-histidine-functionalized rice-straw CNF fluorescent probes also realized sub-parts-per-billion detection of Cr(VI)/Hg(II) [72], showcasing the high-value conversion potential of agricultural waste.

In electrochemical sensing, CNF architectures demonstrate a dual contribution: their interconnected, three-dimensional porosity amplifies electroactive surface area while simultaneously establishing mixed electron–ion pathways that accelerate charge transfer. This synergy is reflected in recent exemplars, where Fe_3_O_4_/CNF-modified carbon paste electrodes pushed 4-chlorophenol detection to 0.5 nM, PANI/CNF/PVA hydrogel coatings on screen-printed gold electrodes delivered limits of detection down to 0.3 µg L^−1^ for Cd^2+^ and Ni^2+^ [84], and xanthine-oxidase-immobilized CNF bioelectrodes enabled real-time assessment of fish freshness across 3–50 µM [85]. Synthesizing the trends compiled in Table 1, a coherent design trajectory emerges: multiscale conductive networks coupled with surface-charge engineering systematically enhance transduction, broadening sensitivity and linearity not only for electrochemical analytes but also for humidity and temperature stimuli; dynamic, dual-network or multivalent crosslinking strategies resolve the classic trade-off between mechanical robustness and strain responsiveness, preserving signal fidelity under large deformation; and in situ growth of functional nanoparticles or metal–organic frameworks within CNF scaffolds adds catalytic, redox, and photonic sites that unlock multidimensional optical–electrochemical readouts. These advances indicate that CNFs are no longer passive, “green” substrates; they are active, tunable platforms in which architecture, interfacial chemistry, and embedded functionality are co-designed to deliver lower detection limits, faster kinetics, and broader modality coverage—thereby charting a clear pathway from laboratory prototypes to application-ready sensing systems.

## 4. Future Research and Prospects

### 4.1. Overcoming Obstacles in Producing CNFs and Fabricating Sensors

High energy demand in mechanical pathways and reagent costs (e.g., TEMPO) are barriers to low-cost, large-scale production. There is a need to optimize pretreatment further and to develop low-cost, efficient, and green processes. Investigated directions include modulating slurry concentration, recycling of reaction media, and application of innovative reactor geometries (e.g., kneaders and twin-screw extruders for TEMPO systems). Consistency of CNF properties—size and surface chemistry—is critical for consistent sensor function. CNF-based sensors exhibit excellent performance in humidity, resistance, and pressure/strain sensing. For instance, capacitive CNF films can achieve a sensitivity of approximately 28 pF/%RH with a response and recovery time of only about 1 s, and their sensitivity can be significantly enhanced when compounded with graphene oxide or carbon nanotubes [86,87]. In contrast, chitosan/chitin sensors also exhibit competitive sensitivity in impedance or QCM modes (approximately 5.8 MΩ/%RH or 58.8 Hz/%RH), but their response and recovery times are slightly longer (ranging from several seconds to tens of seconds) [88,89]. Overall, CNFs exhibit outstanding performance in humidity sensing and mechanical stability, while chitosan/chitin remains competitive in specific application fields. Reproducible distribution of CNFs and conductive fillers in composites is required for the device properties to be reproducible, and firm interlayer adhesion of CNFs and component adhesion to components (the electrodes and substrate) are required for long life in flexible formats. Parallel efforts should be addressed to scalable, high-precision manufacturing processes for CNF-based sensors, including printing and coating technologies. Accordingly, for 2025–2028, targets can be set to achieve a ≥40% reduction in specific energy relative to a 2024 mechanical-grinding baseline of 5–30 kWh·kg^−1^—converging toward ≤3 kWh·kg^−1^ at line scale through high-solid fibrillation combined with enzyme-assisted and DES/microwave pre-swelling—alongside ≥80% solvent/DES recycling and a ≥30% oxidant-dose reduction in TEMPO-type chemistries. Process-control milestones have coefficients of variation ≤10% for fibril width and surface charge across at least five consecutive lots, enabling reproducible filler dispersion, high-yield printing/coating, and stable device performance.

### 4.2. Biocompatibility, Biodegradability, and Recyclability of CNF-Based Materials

CNF-based materials are inherently biocompatible, biodegradable, and recyclable, making them attractive for sustainable sensor applications. The high biocompatibility of CNFs arises from their natural origin and chemical similarity to extracellular matrix components, which minimizes cytotoxicity and adverse biological responses. CNFs are also fully biodegradable under environmental conditions or through enzymatic degradation, leading to minimal ecological impact compared to synthetic polymers. Moreover, CNF-based composites can be recycled or reprocessed through simple physical or chemical methods, such as re-dispersion in water or mild chemical treatments, allowing for material recovery and reducing waste. These properties not only enhance the environmental friendliness of CNF-based devices but also broaden their potential for biomedical and wearable sensor applications.

### 4.3. Ways of Increasing Sensor Performance

An important challenge is achieving high target-analyte selectivity in real-world matrices without interferences, often demanding intricate surface functionalization or the integration of highly selective recognition components. Despite the renewability, flexibility, and tunable interfacial properties of CNFs, their long-term stability and reproducibility remain challenging. CNFs are highly hydrophilic, making them sensitive to environmental humidity; swelling and shrinkage can induce structural changes, interfacial instability, and signal drift, reducing device reliability. Thermal, humidity, and UV exposure can further degrade the hydrogen-bonded network, while microbial activity may compromise device lifespan. In composite systems, weak noncovalent interactions between CNFs and conductive fillers can lead to filler aggregation, interface delamination, and unstable electrical responses over time. Variations in CNF sources and preparation methods result in differences in fibril dimensions, crystallinity, and surface chemistry, causing batch-to-batch performance inconsistency. As CNFs are intrinsically insulating, their conductivity relies on fillers or ionic media, which may degrade via oxidation, migration, or ion leaching during prolonged use. Flexible and wearable devices are also susceptible to mechanical fatigue under repeated bending or stretching. Strategies to improve long-term stability and reproducibility include hydrophobic or crosslinked surface modification, covalent filler–CNF interfaces, standardized CNF synthesis, and protective encapsulation to mitigate environmental effects. Lower detectability limits and higher sensitivity should be possible through ongoing efforts. Another critical focus for the future is the creation of CNF-based platform and array architectures amenable to simultaneous multi-analyte detection. Cross-modal reliability targets can be articulated as follows: humidity responses ≤1.0 s and recoveries ≤3.0 s over 15–90% RH with hysteresis ≤2% RH and 24 h drift ≤1% RH at 50% RH; strain sensing with GF ≥ 10 over 0–50% strain (R^2^ ≥ 0.98), response ≤100 ms, and ≥50,000 cycles at 30% strain with ΔR/R_0_ drift ≤5%; and temperature readouts with TCR ≥ 20 °C^−1^ and response ≤100 ms from −20 to 60 °C. Durability and analytical performance are expected to achieve <5% sensitivity loss after 20 h UV-A and >90% retention after 1000 combined humidity/thermal cycles with thin-film encapsulation, alongside LODs ≤ 0.5 nM for 4-chlorophenol and ≤0.2 µg L^−1^ for Cd^2+^/Ni^2+^ on disposable paper/CNF platforms, while enzyme-based biosensors maintain a ≥14 day operational half-life at 25 °C with ≥80% signal.

### 4.4. Directions for Sustainable and Economically Viable CNF Sensing Technologies

Success relies on greener CNF manufacturing (e.g., enzymatic processes or mild-acid hydrolysis with recyclable acids) and green sensor manufacturing. Greener biodegradability of CNFs can be leveraged for green, disposable sensors for smaller electronic-waste footprints. Looking forward, the development of sustainable and biodegradable sensing substrates will continue to expand the scope of CNF-based technologies. Paper- and cellulose-derived platforms have shown particular promise for green and low-cost electrochemical detection. For instance, Hassan and Fathy recently designed a paper-based potentiometric sensor modified with coumarin derivatives and V_2_O_5_ nanoparticles for selective Pb(II) detection, achieving an ultralow detection limit of 2.1 × 10^−8^ M and a fast response of approximately 8 s. Such advances highlight the future potential of cellulose-based materials as versatile, eco-friendly sensing platforms for environmental monitoring and health diagnostics [90]. Integration with flexible printed electronics will enable wearable and Internet of Things (IoT) applications, but commercial acceptability demands an appropriate balance of price and performance. Exploration of synergies of CNFs and auxiliary nanomaterials (e.g., MXenes, quantum dots, and MOFs) can give rise to new sensing modalities. Successfully, the long-term potential of CNF-based sensors relies on a multidisciplinary plan: enhancing the sustainability and economy of CNF manufacturing; material design for improved selectivity, stability, and sensitivity; and scalable fabrication routes suitable for large-scale manufacturing. The inherent “green” nature of CNFs is a driving force, but economic viability will control market translation; the reliability of sensors will control practical applications; and scalable fabrication will move the field from laboratory demonstrators. Milestones are proposed to operationalize circularity and manufacturability: ≥70% recovery of CNF-based laminates via aqueous re-dispersion or mild delamination, ≥90% mass loss within 12 weeks under controlled composting, roll-to-roll device yields ≥95%, and line-width and device-to-device sensitivity coefficients of variation ≤10% across ≥1000 units per run. At the module level, a materials-cost threshold ≤ USD 0.02 cm^−2^ for humidity/strain patches is envisioned while maintaining the foregoing response, stability, and durability specifications, thereby enabling disposable or recyclable nodes for distributed IoT deployments.

## 5. Conclusions

Cellulose nanofibrils have progressed from a nominally “green” filler to a deliberately engineered platform for intelligent, multimodal sensing, enabled by advances that span high-solid mechanical fibrillation, benign chemical and enzymatic tailoring, and hybrid pretreatments that together yield fibrils with a programmable surface charge, aspect ratio, and colloidal stability; these parameters translate into robust films, aerogels, and double-network hydrogels that convert nanoscale ion, proton, and electron transport into reliable macroscopic signals across humidity, temperature, strain and pressure, optical, electrochemical, and biosensing modalities while remaining compatible with sustainable processing. A coherent design grammar has emerged in which multiscale conductive architectures—leveraging MXenes, silver nanowires, graphene, liquid metals, or redox-active nanoparticles—bridge electronic and ionic pathways to deliver rapid responses and wide dynamic ranges; interfacial charge engineering on oxidized CNF modulates proton and ion migration to elevate sensitivity without sacrificing linearity; and dynamic or dual-network crosslinking through hydrogen bonding, ionic and metal coordination, or supramolecular motifs dissipates mechanical energy under large deformation yet stabilizes electrical readouts over extended duty cycles; collectively, these strategies underpin sub-second humidity sensing, compliant and wide-window strain or pressure monitoring, and nanomolar-level electroanalytical detection, positioning CNFs as high-performance materials rather than a mere sustainable substitute. To convert laboratory momentum into industrial impact, efforts should prioritize three levers: process innovation that lowers embodied energy and reagent burden via high-consistency fibrillation, recyclable media, and enzyme-assisted routes while narrowing distributions in size and surface chemistry to suppress batch variability; manufacturing pathways that adopt printing, coating, and roll-to-roll patterning to ensure uniform filler dispersion, strong interlayer adhesion, and durable interfaces within flexible stacks; and functional hardening that secures selectivity and long-term stability in complex matrices through immobilized bioreceptors, catalytic sites, or porous hosts that resist leaching, alongside arrayed and multimodal nodes for spatially resolved analyte mapping. In parallel, standardized metrology, accelerated ageing, and early-life-cycle assessment should be embedded to de-risk scale-up. Looking ahead, the convergence of green CNF production, algorithm-guided structural design, and tightly coupled “sense–store–compute” architectures—augmented by self-healing, self-powered, and fully degradable device concepts—offers a credible route to wearable and distributed IoT systems in which circularity, cost, and reliability are co-optimized, establishing CNFs as benchmark platforms for next-generation intelligent sensing.

## Figures and Tables

**Table 1:** Bio-based vs. synthetic approaches for flexible and multimodal sensors.

Aspect	Bio-Based Sensor Platforms (e.g., CNFs, Bacterial Cellulose, Chitosan-/Silk-Derived Matrices)	Synthetic Sensor Platforms (e.g., PI/PET/PDMS/PVDF Polymers; Si/Metal-Oxide Microsystems)	Design Implications
Feedstock and sustainability	Renewable biomass; green pretreatments possible (enzymes, DESs); potential for recyclable media and lower embodied burden.	Predominantly petroleum-derived polymers and energy-intensive inorganics.	Bio-based routes reduce e-waste footprint and align with circularity goals; synthetics benefit from mature commodity supply chains.
Intrinsic biocompatibility	Generally high; CNFs and related biopolymers are well-tolerated and biodegradable.	Variable; many require passivation/encapsulation for bio-contact.	Bio-based scaffolds suit wearables/biomedical interfaces; synthetics require additional surface engineering.
Baseline electrical behavior	Insulating backbones; performance arises from ionic/proton transport and percolating conductive networks (MXenes, AgNWs, graphene, liquid metals) engineered within CNFs.	Either insulating (needs fillers) or intrinsically functional (e.g., piezoelectric PVDF; semiconducting oxides).	CNFs leverage surface-charge regulation and network topology to fine-tune sensitivity/linearity; synthetics may offer built-in functions but often at e-waste cost.
Processing and patterning	Aqueous processing; film casting, vacuum filtration, gelation; compatible with printing/roll-to-roll and in situ growth of active phases.	Wide process window from solution casting to photolithography and microfabrication.	CNFs enable low-temperature, solvent-lean routes and direct integration on paper/plastic; synthetics excel in high-temperature microfabrication.
Mechanical profile	High specific strength; compliant films/aerogels/hydrogels; dynamic/dual-network crosslinking dissipates energy while preserving signal fidelity under strain.	Broad: elastomers (PDMS) to high-Tg films (PI); robust thermal profiles.	CNF architectures can match flexibility while offering tunable mechanics via hydrogen bonds/ionic/metal coordination.
Environmental stability	Hydrophilic; prone to humidity-driven swelling and signal drift without encapsulation/chemical stabilization.	Typically better moisture/thermal stability out of the box.	CNFs demand encapsulation, hydrophobization, or covalent filler interfaces for long-term stability; synthetics suit harsher ambient conditions.
Modalities demonstrated	Humidity, temperature, strain/pressure, optical, electrochemical, biosensing with sub-second humidity response and nanomolar electroanalysis achievable in CNF composites.	Broad coverage across the same modalities; extensive legacy in micro- and opto-electronics.	CNFs are no longer a “green substrate” only—they are active, tunable platforms across modalities when networks and interfaces are co-designed.
Representative device performance (from this review)	Humidity: <1 s response (CNF/MXene/AgNWs); printed CNF–AgNPs humidity with fast recovery; strain/temperature hydrogels functional at sub-zero; electrochemical detection to nM–µM regimes.	Not focus of this review; included here for context.	CNF composites already meet or approach the state of the art for many use cases while offering sustainability dividends.
End of life	Biodegradable/recyclable; opportunity to curb e-waste (≈62 Mt in 2022) with degradable/compostable form factors.	Persistent; recycling varies by polymer; inorganic residues remain.	CNFs enable green, disposable, or recyclable sensors for distributed IoT and packaging.
Key risks/mitigations	Batch variability, humidity sensitivity, filler aggregation; mitigate via standardized CNF synthesis, encapsulation, covalent filler bonding.	Long-established QC; environmental burden at the end of life.	A clear materials–process–architecture playbook exists to harden CNF devices for deployment.

**Table 2:** Comparison of different types of CNF-based functional sensors.

Sensor Type	Material System/Composition	Structural Form	Detected Performance	References
Humidity sensor	TOCNF/MXene/AgNWs	Composite nanopaper	The RH range is wide, with a reversible response and a recovery time of 185 s under NIR.	[60]
Humidity sensor	CNF/AgNPs	Inkjet-printing film	35–70% RH; high sensitivity response 4 s/recovery 43 s.	[61]
Humidity sensor	TOCNFs (surface charge regulation)	Thin-film type	15–75% RH; strong linearity, sensitivity 44.5% (%RH).	[62]
Humidity sensor	CNF/Liquid Metal/Gelatin/Graphene Oxide	Antifreeze hydrogel	Response 210 ms/recovery 150 ms.	[63]
Temperature and pressure sensor	PVA/CNF@TA/AgNPs	Multifunctional flexible membrane	High sensitivity (GF ≈ 46.42), low detection limit (<1%), response time of 80 milliseconds; temperature coefficient change rate (TCR ≈ 29.84/°C).	[64]
Pressure sensor	Micro/Nano Cellulose/Silk Fibroin/Graphene Paper (flexibility/strength)	Composite paper	High sensitivity (GF up to 2.31), wide detection range, and excellent stability.	[66]
Pressure sensor	Carboxylated CNF/Tannic Acid/PVA/Al3+	Hydrogel	After electrode spacing optimization, it exhibited a high sensitivity of 0.0654 log(ΔR/%RH), with response and recovery times of approximately 4 s and 34 s, respectively.	[67]
Pressure sensor	TOCNF/Polyacrylamide/LiCl/Glycerol	Dual-network hydrogel	Sensitivity (GF value up to 8.6), response time approximately 290 ms.	[68]
Biosensor	TOCNF/CGG/DADA-AuNPs	Paper-based	The test can detect 10 CFU/mL within 15 min.	[70]
Biosensor	TOCNF/Glucose Oxidase/Phenol Red	Hydrogel	Detect glucose concentration of 7–13 mM at 440 °C.	[71]
Optical sensor	L-histidine-modified CNFs	Fluorescent sensing probe	Detectable Cr, Hg; low detection limit.	[72]
Optical sensor	TEMPO-CNF/HCl	Aerogel	Visible structural color changes can be induced by an external force as low as 0.05 N; the response time is approximately 0.3 s.	[73]
Optical sensor	TOCNF/Eu-MOF	Thin-film type	Response time within 30 s; detection can reach 1.27 μM.	[74]
Optical sensor	ACNF/DIES	Thin-film type	Linear fluorescence response in the pH range of 3–11.	[75]
Electrochemical sensor	Fe3O4/CNF/CPE	Carbon paste electrode	Accurate detection is achieved within a linear range (e.g., 0.01–100 μM), with a detection limit as low as the nanomolar level (~6.3 nM), and a response time of just a few seconds.	[81]
Electrochemical sensor	PANI/CNF/PVA/SPGE	Hydrogel	Exhibits a good linear response relationship to Cd2+ and Ni2+ with extremely low detection limits (0.24 μg/L and 0.31 μg/L, respectively).	[84]
Electrochemical sensor	CNF/GCE/XO	Hydrogel film	The linear detection range is 3–50 μM, with a detection limit of 47.96 nM (approximately 4.8 × 10−8 M), and a sensitivity as high as 5281 μA mM−1 cm−1; the response time is between 4 and 10 s.	[85]

## Data Availability

No new data were created or analyzed in this study. Data sharing does not apply to this article.
